# The intention of Egyptian healthcare workers to take the monkeypox vaccine: is urgent action required?

**DOI:** 10.1186/s12913-024-11147-0

**Published:** 2024-10-08

**Authors:** Ramy Mohamed Ghazy, Mai Hussein, Shymaa Mamdouh Mohamed Abdu, Doha El-sayed Ellakwa, Mahmoud M. Tolba, Naglaa Youssef, Amira Saad Mahboob, Samar Abd ElHafeez

**Affiliations:** 1 Tropical Health, High Institute of Public Health, Alexandria University, Alexandria, Egypt; 2 Alexandria Clinical Research Administration, Alexandria Health Affairs Directorate, Alexandria, Egypt; 3 Ministry of Health and Population, Cairo, Egypt; 4 Public Health and Community Medicine Department, Faculty of Medicine, Mansoura University, Mansoura, Egypt; 5 Biochemistry Department Faculty of Pharmacy, Al-Azhar University, Cairo, Egypt; 6 Department of Biochemistry, Faculty of Pharmacy, Sinai University, Kantara Branch, Ismailia, Egypt; 7 Pharmaceutical Division, Ministry of Health and Population, Cairo, Egypt; 8 Medical-Surgical Nursing Department, Faculty of Nursing, Cairo University, Cairo, Egypt; 9 Department of Occupational Health and Industrial Medicine, High Institute of Public Health, Alexandria University, Alexandria, Egypt; 10 Epidemiology Department, High Institute of Public Health, Alexandria University, Alexandria, Egypt

**Keywords:** Monkeypox, Vaccine hesitancy, Emerging diseases, Healthcare worker, Egypt

## Abstract

**Background:**

In light of the ongoing monkeypox (MPOX) epidemic, healthcare workers (HCWs) have been in contact with various diseases. Therefore, they should take appropriate preventive and control measures to maintain their health. This study assessed Egyptian HCWs’ intentions to take MPOX vaccines.

**Methods:**

A cross-sectional survey was conducted using social media platforms between September 27 and November 4, 2022. An anonymous online survey using the 5C scale was conducted using convenience and snowball sampling methods to assess the five psychological antecedents of vaccination (i.e., confidence, constraints, complacency, calculation, and collective responsibility).

**Results:**

A total of 399 HCWs with a mean age of 32.6 ± 5.7 participated in this study. Of them, 89.7% were female. The five C psychological antecedents of vaccination were as follows: 55.9% were confident about vaccination, 50.6% were complacent, 56.6% experienced constraints, 60.7% calculated the risk and benefit, and 58.4% had collective responsibility. Multivariate analysis showed that high income level and having information about MPOX were significant predictors of confidence in the MPOX vaccines (adjusted odds ratio ((AOR) = 4.19, 95% CI (1.12– 15.59), *P* = 0.032). Participants aged 31–45 years and 19–30 years showed significant association (AOR = 2.46, 95% CI (0.85–7.15), *P* = 0.096) and (AOR = 4.19, 95% CI (1.39–12.64), *P* = 0.011), respectively. Having an idea about the MPOX vaccines significantly predicted the complacency domain (AOR = 3.77, 95%CI (1.47–9.65, *P* = 0.006). Moreover, precollege/undergraduate education and having an idea about MPOX vaccination were significant predictors of the constraint domain (AOR = 1.81.95% CI (1.09–2.99, *P* = 0.020), (AOR = 2.70, 95% CI (1.05–6.95, *P* = 0.038), respectively). Female sex, having a diploma, postgraduate studies, and having an idea about MPOX vaccine significantly predicted calculation domain (AOR = 2.06, 95% CI (1.05–4.04, *P* = 0.035), (AOR = 3.98,95% CI (1.33–11.87, *P* = 0.013), (AOR = 2.02, 95% CI (1.25–3.26, *P* = 0.004) & (AOR = 2.75. 95% CI (1.05–7.18, *P* = 0.039), respectively. The only significant predictor of collective responsibility was having a diploma and postgraduate studies (AOR = 3.44, 95% CI (1.21–9.78, *P* = 0.020), (AOR = 1.90,95% CI (1.17–3.09, *P* = 0.009).

**Conclusions:**

Efforts to control MPOX should focus on promoting protective measures such as the vaccination of HCWs as well as raising their awareness about the updated information regarding the virus and the approved vaccines.

**Supplementary Information:**

The online version contains supplementary material available at 10.1186/s12913-024-11147-0.

## Introduction

Monkeypox (MPOX) is a zoonotic infectious disease caused by the monkeypox virus (MPOXV), a prominent double-stranded DNA virus belonging to the Orthopoxvirus genus and family [1]. The virus was first discovered in 1958 after outbreaks in research-held monkeys exhibited symptoms resembling small pox [2]. In 1970, MPOXV was identified in humans when infants in the Democratic Republic of the Congo were initially misdiagnosed with smallpox, and later outbreaks occurred outside of Africa, including the United States in 2003 [3, 4].

Recognizing the global threat posed by MPOX, the World Health Organization (WHO) designated it as an emerging disease in 2018, emphasizing the need for research and development in this area [5]. The ongoing worldwide outbreak of MPOX has been classified as a Public Health Emergency of International Concern, underscoring the urgency of effective prevention and control strategies [6]. While specific treatments such as tecovirimat, brincidofovir, and vaccinia immunoglobulin have been licensed for MPOX treatment, vaccination remains the most effective means of preventing and controlling infectious diseases, including MPOX [1, 7–9].

Healthcare workers (HCWs) play a crucial role in disease surveillance, diagnosis, and management, making their vaccination against MPOX essential [10]. However, vaccine hesitancy (VH) among HCWs has emerged as a significant challenge, influenced by psychological factors and beliefs surrounding vaccination [11–13]. Understanding the attitudes and concerns of HCWs regarding MPOX vaccination is vital for addressing VH and ensuring the effective implementation of vaccination programs.

The 5C scale is a tool developed to improve the effectiveness of measures that influence an individual’s decision to get vaccinated. Unlike other tools that only consider the 3 C model, which includes confidence, complacency, and constraints, the 5C scale evaluates five psychological antecedents. These include confidence in the vaccine’s safety and efficacy, complacency towards essential risk factors, constraints related to logistical capacity, calculation of available medical information, and collective responsibility for public health [14]. The 5C scale has already been used extensively to measure VH towards seasonal influenza vaccine [15], coronavirus diseases 2019 (COVID–19) [16], and monkeypox [17, 18].

In the context of the ongoing MPOX epidemic, HCWs are likely to come into contact with the disease and should take appropriate preventive and control measures [10]. However, the attitudes of HCWs towards MPOX vaccination in Egypt have not been extensively studied. Therefore, this study aimed to explore the potential psychological antecedants of Egyptian HCWs if the MPOX vaccine were to be mandated. By examining HCWs’ perceptions, concerns, and beliefs, we can identify factors that may contribute to VH and develop targeted interventions to address these issues. Ultimately, this research aimed to contribute to the improvement of vaccination acceptance and uptake among HCWs in Egypt, enhancing overall public health outcomes.

## Materials and methods

### Study design

An online cross-sectional survey was conducted anonymously via commonly used social media platforms, including Facebook, WhatsApp, and Twitter, between September 27 and November 4, 2022.

### Study population and sample size

The individuals considered for the study were HCWs of 18 years or above who reside in Egypt. We presumed that 50% of the HCWs would be willing to receive the MPOX vaccine due to the lack of prior research on their attitudes towards it in Egypt. The required sample size for the investigation was calculated using the following formula: *n* = Z^2^ 1 − α/2P (1 − P)/e2. Thus, P is the estimated prevalence of VH, n is the minimum number of respondents required, Z^2^ is the relative value of 1.96 for the 95% confidence interval (CI), and e is the necessary accuracy of 5%. According to our calculations, the study required a sample size of at least 384 HCWs. To account for any inconsistent or incomplete data, we increased the sample size to 400 participants. We distributed the survey using convenience and snowball sampling methods, and shared the questionnaire link through social and work groups of HCWs across the country.

### Data collection tool

The questionnaire’s initial part provided information about the study objectives, requested consent to participate, and guaranteed confidentiality of responses.

 The survey consisted of several sections, including a sociodemographic characteristics section that covered age, gender, nationality, living area, self-reported financial status (categorized as low, middle, or high income), residence, level of education, marital status, occupation, and presence of comorbidities. This section also had two yes/no questions asking if the participants had ever been sick with MPOX and whether they were aware of different MPOX vaccinations. Section 2 had 15 questions covering 5Cs: confidence, complacency, constraints, calculation, and collective responsibility. Participants rated on a scale of 1 (strongly disagree) to 5 (strongly agree). The 5C questionnaire was designed to evaluate the attitudes and beliefs of the participants concerning MPOX vaccination. This tool had been previously validated in Arabic in a separate study [19]. The cutoff points for each domain were established as well [20, 21]. Both English and Arabic versions were provided for participants to select the most appropriate for them (Supplementary file). Participants could only submit one response per IP address to ensure a single entry. The survey’s opening page included information on the study’s research goals, participation consent, and guarantees of anonymity. The allotted time for responding to the questionnaire was 5 to 10 min.

### Operational definitions

#### Confidence

This term refers to people’s confidence in vaccination, including their belief in its dependability and efficacy [22], as well as in the healthcare system and HCWs. The adoption of vaccines may diminish when there is a lack of trust or mistrust, which may result in a loss of faith in the healthcare system and an increase in the acceptance of false information. The survey’s confidence domain questions were (1) I have absolute confidence that vaccines are safe (2), I have absolute confidence that vaccinations work, and (3) I have absolute confidence that public authorities will make vaccine decisions that are in the best interests of the community [19, 23].

Constraints include structural and psychological impediments, such as access, time, self-efficacy, empowerment, and a lack of behavioral control that may prevent people from receiving vaccinations [23]. Even if an individual intends to receive the vaccine, these barriers may prevent them from doing so. Questions in the constraints domain of the survey asked about the impact of everyday stress on vaccination, the inconvenience of receiving vaccines, and discomfort with visiting doctors [19, 23].

#### Complacency

When people are complacent, they view the risks of vaccine-preventable diseases as minimal and do not view vaccination as a required preventive measure [21]. The perception that vaccine-preventable diseases are uncommon, the idea that a healthy immune system may provide sufficient protection, and the concept that vaccine-preventable diseases are not severe enough to merit vaccination were also addressed in the complacency area of the survey [19, 23].

Calculation refers to gathering data to contrast the risks of contracting a disease by receiving vaccination to make an informed choice [19]. This behavior, interpreted as an indication of risk aversion, may be detrimental to vaccination practices. The survey’s calculation domain questions covered topics such as balancing benefits and dangers, carefully examining the value of each vaccination, and the significance of comprehending the basics of vaccination before receiving it [19, 23].

The willingness to use vaccination to protect others by boosting herd immunity is referred to as collective responsibility [24]. It describes people who vaccinate themselves to protect others and reduce the spread of disease. The survey’s collective responsibility domain questions covered the idea that vaccination is a collaborative effort to stop the spread of disease, that getting vaccinated can also protect those with weakened immune systems, and that when everyone is vaccinated, individuals are not required to be vaccinated [19, 23].

### Statistical analysis

SPSS) version 21.0 was used to conduct the statistical analysis. The participants’ demographic information and their responses on the 5C scale were summarized using descriptive statistics in the form of numbers and percentages. The association between the independent variables (respondents’ sociodemographic traits and attitudes toward MPOX vaccination) was evaluated using bivariate analysis. Multivariate logistic regression analysis was used to identify the factors influencing the decision to receive MPOX vaccines. Factors with a *P*-value of less than 0.15 in the bivariate analysis were included in the regression analysis. The variables were described using adjusted odds ratios (OR) and 95% confidence intervals (CI), with *P*-values less than 0.05 regarded as statistically significant. To estimate each predictor’s contribution to the results of the multivariate analysis, coefficients were calculated for each predictor in the final model while accounting for the other model variables. The overall model fit was evaluated using the likelihood ratio test and omnibus test, and all five fitted models performed better than the null model (*P* < 0.05). The variation in the dependent variable described by each model is shown by the Cox and Snell R-square and the Nagelkerke R-square, respectively. The Wald chi-square test (*P* < 0.05) was used to determine whether the specific regression coefficients (ß) were statistically significant. The Hosmer-Lemeshow test was performed to determine whether the predicted and observed probabilities matched, with *P*-values higher than 0.05, indicating a positive result.

### Ethics

This study was approved by the Research Ethics Committee of the High Institute of Public Health, Alexandria University (IRB No. 00012098/FWA No. 00018699). The study was performed in accordance with the relevant guidelines and regulations, that is Declaration of Helsinki. All participants gave their informed consent before completing the questionnaire, as displayed on the electronic questionnaire cover page. Participants were provided with clear explanations of the study’s objectives, procedures, and potential risks and benefits. The survey guaranteed participants that their responses would be kept confidential.

### Privacy and confidentiality

To safeguard participant anonymity and privacy, unique identification codes were assigned to individuals, and no personally identifiable information was gathered. Data storage adhered to industry best practices, with information securely stored on password-protected servers. Rigorous anonymization and aggregation procedures were implemented to prevent individual responses from being linked to specific participants.

## Results

### Characteristics of studied Egyptian healthcare workers

The study sample had a mean age of 32.6 ± 5.7 years. The majority of participants were female (89.7%, *n* = 358), lived in urban areas (92.2%, *n* = 368), were married (65.4%, *n* = 261), had postgraduate degrees (69.2%, *n* = 276), and had middle incomes (83.0%, *n* = 331). Additionally, most participants did not know anyone died due to MPOX (95.7%, *n* = 382) and 85% (*n* = 339) did not have any chronic diseases. A small number of study participants had confirmed MPOX infection (0.8%, *n* = 3), and a few of them were unaware of the various types of MPOX vaccines available (6.8%, *n* = 27) (Table 1).

**Table 1:** Characteristics of the study sample (*n*=399)

**Variables **	**N (%)**
**Age** (Mean± SD)	32.6 ± 5.7
**Gender**	
Male	41 (10.3)
Female	358 (89.7)
**Residence**	
Urban	368 (92.2)
Rural	31 (7.8)
**Marital status**	
Single	134 (33.6)
Married	263 (65.9)
Widow	2 (0.5)
**Income level**	
Low	26 (6.5)
Middle	331 (83.0)
High	42 (10.5)
**Highest educational level**	
Precollege/High school	2 (0.5)
Undergraduate (Bachelor)	99 (24.8)
Diploma	22 (5.5)
Postgraduate (master)	223 (55.9)
Postgraduate (PhD)	53 (13.3)
**Chronic diseases**	
Yes	60 (15)
No	339 (85)
**Having monkeypox**	
Yes	3 (0.8)
No	383 (96.0)
Not sure	13 (3.2)
**Know anyone died from monkeypox**	
No	382 (95.7)
Not sure	17 (4.3)
**Having any idea about various types of monkeypox vaccines**	
Yes	27 (6.8)
No	372 (93.2)

### Egyptian HCWs’ perception toward MPOX vaccines

**Fig. 1:**
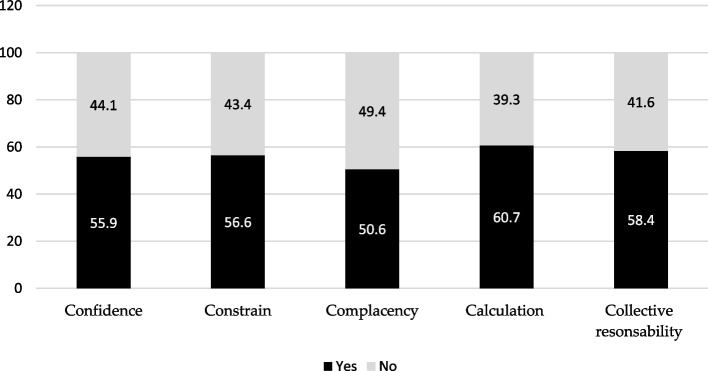
Egyptian HCWs’ perception toward MPOX vaccines using 5C domains

Figure 1; Table 2 display the scores of the 5C participants along with the associated factors. Regarding the confidence domain, over half of the participants (55.9%, *n* = 223) showed confidence in MPOX vaccination. Out of all the participants, those between the ages of 46–63 (63.2%, *n* = 12) and with a high-income level (69%, *n* = 29) appeared to be more confident, but the difference was not statistically significant (*P* = 0.693 and *P* = 0.084, respectively). HCWs without MPOX were more confident than those with MPOX, but the difference was not statistically significant (56.9% vs. 43.1%, *P* = 0.050). More than half of the participants (56.6%, *n* = 226) experienced constraints regarding the MPOX vaccination. Participants aged 19–30 years, attended pre-college/high school/undergraduate (bachelor), and had information about MPOX vaccine significantly affected the constraints domain (*P* = 0.027, *P* = 0.012, and *P* = 0.022, respectively). Nearly half (50.6%, *n* = 202) of the participants were complacent toward a vaccine. HCWs aged 19–30 years (61%, *n* = 72) and those who had an idea about the MPOX vaccine (77.8%, *n* = 21) were significantly more complacent than others (*P* < 0.05).

**Table 2:** Factors associated with the domains of the 5C scale among Egyptian HCWs (*n* = 399)

Variables	Confidence		Complacency		Constraints		Calculation		Collective responsibility	
	Yes	No	Yes	No	Yes	No	Yes	No	Yes	No
	*n* (%)	*n* (%)	*n* (%)	*n* (%)	*n* (%)	*n* (%)	*n* (%)	*n* (%)	*n* (%)	*n* (%)
**Age (in years)**										
19–30	68 (57.6%)	50 (42.4%)	72 (61%)	46 (39%)	77 (65.3%)	41 (34.7%)	71 (60.2%)	47 (39.8%)	69 (58.5%)	49 (41.5%)
31–45	143 (54.6%)	119 (45.4%)	125 (48.7%)	137 (52.3%)	142 (54.2%)	120 (45.8%)	158 (60.3%)	104 (39.7%)	150 (57.3%)	112 (42.7%)
46–63	12 (63.2%)	7 (36.8%)	5 (26.3%)	14 (73.7%)	7 (36.8%)	12 (63.2%)	13 (68.4%)	6 (31.6%)	14 (73.7%)	5 (26.3%)
**Test, ** ***p*** **-value**	0.734, 0.693		**10.481, 0.005** ^a^		**7.234, 0.027** ^a^		0.505, 0.777		1.969,0.374	
**Gender**										
Male	22 (53.7%)	19 (46.3%)	21 (51.2%)	20 (48.8%)	23 (56.1%)	18 (43.9%)	19 (46.3%)	22 (53.7%)	23 (56.1%)	18 (43.9%)
Female	201 (56.1%)	157 (43.9%)	181 (50.6%)	177 (49.4%)	203 (56.7%)	155 (43.3%)	223 (62.3%)	135 (37.7%)	210 (58.7%)	148 (41.3%)
**Test, ** ***p*** **-value**	0.092, 0.761		0.006, 0.936		0.006, 0.941		**3.921, 0.048** ^a^		0.099, 0.753	
**Residence**										
Urban	206 (56%)	162 (44%)	182 (49.55)	186 (50.5%)	206 (56%)	162 (44%)	224 (60.9%)	144 (39.1%)	217 (59%)	151 (41%)
Rural	17 (54.8%)	14 (45.2%)	20 (64.5%)	11 (35.5%)	20 (64.5%)	11 (35.5%)	18 (58.1%)	13 (41.9%)	16 (51.6%)	15 (48.4%)
**Test, ** ***p*** **-value**	0.015, 0.902		2.594, 0.107		0.849, 0.357		0.094, 0.759		0.637, 0.425	
Marital status										
Single/widow	75 (54.3%)	63 (45.7%)	73 (52.9%)	65 (47.1%)	86 (62.3%)	52 (37.7%)	82 (59.4%)	56 (40.6%)	80 (58%)	58 (42%)
Married	148 (56.7%)	113 (43.3%)	12 (49.4%)	132 (50.6%)	140 (53.6%)	121 (46.4%)	16 (61.3%)	101 (38.7%)	153 (58.6%)	108 (41.4%)
**Test, ** ***p*** **-value**	0.203, 0.652		0.436, 0.509		2.769, 0.096		0.134, 0.714		0.016, 0.900	
**Income level**										
Low	11 (42.3%)	15 (57.7%)	12 (46.2%)	14 (53.8%)	15 (57.7%)	11 (42.3%)	11 (42.3%)	15 (57.7%)	17 (65.4%)	9 (34.6%)
Middle	183 (55.3%)	148 (44.7%)	172 (52%)	159 (48%)	184 (55.6%)	147 (44.4%)	206 (62.2%)	125 (37.8%)	196 (59.2%)	135 (40.8%)
High	29 (69%)	13 (31%)	18 (42.9%)	24 (57.1%)	27 (64.3%)	15 (35.7%)	25 (59.5%)	17 (40.5%)	20 (47.6%)	22 (52.4%)
**Test, ** ***p*** **-value**	4.944, 0.084		1.459, 0.482		1.160, 0.560		4.036, 0.133		2.622, 0.270	
**Highest educational level**										
Precollege/High school/ Undergraduate (Bachelor)	59 (58.4%)	42 (41.6%)	56 (55.4%)	45 (44.6%)	70 (69.3%)	31 (30.7%)	48 (47.5%)	53 (52.5%)	48 (47.5%)	53 (52.5%)
Diploma	16 (72.7%)	6 (27.3%)	12 (54.5%)	10 (45.5%)	12 (54.5%)	10 (45.5%)	17 (77.3%)	5 (22.75)	16 (72.7%)	6 (27.3%)
Postgraduate studies	148 (53.6%)	128 (46.4%)	134 (48.6%)	142 (51.4%)	144 (52.2%)	132 (47.8%)	177 (64.1%)	99 (35.9%)	169 (61.2%)	107 (38.8%)
**test statistics, ** ***p*** **-value**	3.366, 0.186		1.549, 0.461		**8.880, 0.012** ^a^		**11.239, 0.004** ^a^		**7.687, 0.021** ^a^	
**Chronic diseases**										
Yes	33 (55%)	27 (45%)	26 (43.3%)	34 (56.7%)	35 (58.3%)	25 (41.7%)	38 (63.3%)	22 (36.7%)	39 (65%)	21 (35%)
No	190 (56%)	149 (44%)	176 (51.9%)	163 (48.1%)	191 (57.3%)	148 (43.7%)	204 (60.2%)	135 (39.8%)	194 (57.2%)	145 (42.8%)
**Test, ** ***p*** **-value**	0.023, 0.880		1.503, 0.220		0.082, 0.774		0.213, 0.645		1.268, 0.260	
**Had confirmed monkeypox infection**										
Yes	2 (66.7%)	1 (33.3%)	1 (33.3%)	2 (66.7%)	1 (33.3%)	2 (66.7%)	3 (100%)	0 (0%)	2 (66.7%)	1 (33.3%)
No	218 (56.9%)	165 (43.1%)	193 (50.4%)	190 (49.6%)	217 (56.7%)	166 (43.3%)	233 (60.8%)	150 (39.2%)	223 (58.2%)	160 (41.8%)
Not sure	3 (23.1%)	10 (76.9%)	8 (61.5%)	5 (38.5%)	8 (61.5%)	5 (38.5%)	6 (46.2%)	7 (53.8%)	8 (61.5%)	5 (38.5%)
**test statistics, ** ***p*** **-value**	5.983, 0.050 ^a^		0.987, 0.611		0.791, 0.673		3.097, 0.213		0.142, 0.931	
**Know anyone who died from monkeypox**										
No	215 (56.3%)	167 (43.7%)	193 (50.5%)	189 (49.5%)	218 (57.1%)	164 (42.9%)	231 (60.5%)	151 (39.5%)	221 (57.9%)	161 (42.1%)
Not sure	8 (47.1%)	9 (52.9%)	9 (52.9%)	8 (47.1%)	8 (47.1%)	9 (52.9%)	11 (64.7%)	6 (35.3%)	12 (70.6%)	5 (29.4%)
**Test, p-value**	0.562, 0.454		0.038, 0.845		0.664, 0.415		0.122, 0.727		1.086, 0.297	
**Having an idea about the types of monkeypox vaccines**										
Yes	19 (70.4%)	8 (29.6%)	21 (77.8%)	6 (22.2%)	21 (77.8%)	6 (22.2%)	21 (77.8%)	6 (22.2%)	18 (66.7%)	9 (33.3%)
No	204 (54.8%)	168 (45.2%)	181 (48.7%)	191 (51.3%)	205 (55.1%)	167 (44.9%)	221 (59.4%)	151 (40.6%)	215 (57.8%)	157 (42.2%)
**Test, ** ***p*** **-value**	2.463, 0.117		**8.541, 0.003** ^a^		**5.268, 0.022** ^a^		3.559, 0.059		0.815, 0.367	

^a^Significant, Chi-square test

Regarding the calculation, 60.7% (*n* = 242) of the participants calculated the risks and benefits of the vaccine. A total of 62.3% (*n* = 223) of female participants and 64.1% (*n* = 177) of postgraduate participants significantly calculated the risks and benefits of the MPOX vaccine (*P* = 0.048 and 0.004, respectively).

Among the participants, 58.4% (*n* = 223) felt they had a greater responsibility towards the vaccine. The healthcare workers with diplomas (72.7%, *n* = 16) and postgraduate studies (61.2%, *n* = 169) felt a significantly higher collective responsibility towards MPOX vaccination than other participants (*P* = 0.021).

### Multivariate analysis of factors associated with the 5C scale

Multivariate analysis showed that high income level and having information about MPOX were significant predictors of confidence in the MPOX vaccines among Egyptian HCWs (adjusted odds ratio ((AOR) = 4.19, 95% CI (1.12– 15.59), *P* = 0.032). Participants aged 31–45 years and 19–30 years showed significant association with complacency (AOR = 2.46, 95% CI (0.85–7.15), *P* = 0.096) and (AOR = 4.19, 95% CI (1.39–12.64), *P* = 0.011), respectively.

In our sample of Egyptian HCWs, having an idea about the MPOX vaccines significantly predicted the complacency domain (AOR = 3.77, 95%CI (1.47–9.65), *P* = 0.006). Moreover, precollege/undergraduate education and having an idea about MPOX vaccination were significant predictors of the constraint domain (AOR = 1.81, 95% CI (1.09–2.99), *P* = 0.020), (AOR = 2.70, 95% CI (1.05–6.95), *P* = 0.038), respectively. Female sex, having a diploma, postgraduate studies, and having an idea about MPOX vaccine significantly predicted the calculation domain (AOR = 2.06, 95% CI (1.05–4.04), *P* = 0.035), (AOR = 3.98, 95% CI (1.33–11.87), *P* = 0.013), (AOR = 2.02, 95% CI (1.25–3.26), *P* = 0.004) & (AOR = 2.75, 95% CI (1.05–7.18), *P* = 0.039), respectively. The only significant predictor of collective responsibility were having a diploma and postgraduate studies (AOR = 3.44, 95% CI (1.21–9.78), *P* = 0.020), (AOR = 1.90, 95% CI (1.17–3.09), *P* = 0.009) (Table 3).

**Table 3:** Univariate and multivariate analyses of predictors of 5C scale domains among Egyptian healthcare workers (*n* = 399)

**5C domains**	**Univariate analysis**		**Multivariate analysis**		
**Confidence**	**COR** ^a^	*P*	**β**	**AOR** ^a^ ** (95% CI)**	***p***
**Income level**					
Low	(r)^a^			(r)^a^	
Middle	1.68(0.75-3.78)	0.201	0.474	1.60(0.71-3.63)	0.254
High	3.04(1.10-8.40)	0.029*	1.081	2.94(1.05-8.21)	0.038*
**Had confirmed monkeypox infection**					
Yes	6.66(0.43-101.73)	0.214	1.919	6.81(0.44-104.43)	0.168
No	4.40(1.19-16.25)	0.016*	1.434	4.19(1.12-15.59)	0.032*
Not sure	(r)^a^			(r)^a^	
**Complacency**					
** Age (years)**					
19-30	4.38(1.47-12.98)	0.004*	1.44	4.19(1.39-12.64)	0.011*
31-45	2.55 (0.89-7.29)	0.056	0.90	2.46(0.85-7.15)	0.096
46-63	(r)^a^			(r)^a^	
** Having an idea about the monkeypox vaccine**					
No	(r)^a^			(r)^a^	
Yes	3.69(1.45-9.35)	0.003*	1.32	3.77(1.47-9.65)	0.006*
**Constraints**					
** Highest educational level**					
Precollege/high school/Undergraduate (Bachelor)	2.06(1.27-3.35)	0.002*	0.59	1.81(1.09-2.99)	0.020*
Diploma	1.10(0.46-2.63)	0.830	0.13	1.14(0.47-2.76)	0.764
Postgraduate studies	(r)^a^			(r)^a^	
** Having an idea about vaccines **					
No	(r)^a^			(r)^a^	
Yes	2.85(1.12-7.22)	0.021*	0.99	2.70(1.05-6.95)	0.038^*^
**Calculation**					
** Gender**					
Male	(r)^a^			(r)^a^	
Female	1.91(0.99-3.66)	0.047*	0.72	2.06(1.05-4.04)	0.035*
** Highest educational level**					
Precollege/high school/undergraduate (Bachelor)	(r)^a^			(r)^a^	
Diploma	3.75(1.28-10.95)	0.011*	1.38	3.98(1.33- 11.87)	0.013*
Postgraduate studies	1.87(1.24-3.13)	0.003*	0.70	2.02(1.25-3.26)	0.004*
** Having an idea about the vaccine**					
No	(r)^a^			(r)^a^	
Yes	2.39(0.94-6.06)	0.059	1.01	2.75(1.05-7.18)	0.039
**Collective responsibility**					
** Highest educational level **					
Precollege/high school/undergraduate	(r)^a^			(r)^a^	
Diploma	2.94(1.06-8.13)	0.032*	1.23	3.44(1.21-9.78)	0.020*
Postgraduate studies	1.74(1.10-2.76)	0.017*	0.64	1.90(1.17-3.09)	0.009*

^a^ *r* Reference group, *COR* Crude odds ratio, *AOR* Adjusted odds ratio

*Significant

## Discussion

VH is a critical phenomenon that endangers human health. It has been recently reported at high rates for COVID–19 and MPOX among adults and children, regardless of their health status specifically in the Middle Eastern region [25–27]. HCWs are at a higher risk of contracting the disease because of their front-line role in caring for sick patients, making it imperative to assess their attitudes regarding MPOX vaccination to prevent the spread of the disease [28]. The opinions of HCWs regarding vaccination may affect the advice they give to patients, how they handle outbreaks, and how they educate the public. While negative attitudes can discourage vaccination and continue the development of MPOX, positive attitudes can promote vaccination uptake and increase the effectiveness of preventive efforts [29]. 

In the current study, we sought to understand the psychological influence of MPOX vaccination among HCWs in Egypt. The results showed that our participants had a negative attitude towards MPOX vaccination. Almost two-thirds (55.9%) had faith in the vaccine’s efficacy, but 50.6% were complacent regarding the dangers of the disease. In addition, 60.7% of our sampled Egyptian HCWs acknowledged the advantages and disadvantages of vaccination, demonstrating a positive attitude towards MPOX vaccination and a sense of shared responsibility for halting the spread of the disease. Moreover, a substantial percentage of Egyptian HCWs (56.6%) reported constraints related to MPOX vaccination such as access and cost issues.

Despite the proven MPOX vaccines’ effectiveness and safety [30], previous studies have shown a noticeable gap in the general attitude of HCWs towards MPOX vaccination. An earlier study from Indonesia showed that 93.6% of general practitioners were willing to receive a smallpox vaccine to protect against MPOX [31]. A less favorable attitude toward MPOX vaccination among HCWs was reported in the Czech Republic (8.8%) [32]. Also, 58.3% of Ghanaian HCWs, between 50% and 60% of the HCWs from the United States, [33] 52.7% of Saudi Arabia HCWs, [34] 55.4% of HCWs in France, [35] and 58.6% of HCWs in Italy [36] showed intention to receive MPOX vaccine, while the majority of Chinese HCWs (90.12%) were hesitant to deliver the MPOX [37].

Conversely, only 31.11% of Nigerian HCWs expressed trust in MPOX vaccination, 58.40% displayed indifference towards MPOX vaccinations, and 63.80% detected constraints in MPOX vaccination [38]. Only 27.2% of Nigerian HCWs considered the advantages and disadvantages of vaccination and 39.2% consented to the MPOX vaccine to protect others. Therefore, Nigerian HCWs exhibited a less positive attitude towards MPOX vaccination than the findings from our study.

Notably, these findings may be specific to the context of each country and cannot be generalized to other countries or regions. VH is a complex issue influenced by various factors, including cultural beliefs, historical experiences, and access to information [22].

Our study found that Egyptian HCWs with higher income levels and those who did not receive MPOX were associated with increased confidence in MPOX vaccination. Younger Egyptian HCWs and those with previous knowledge of the MPOX vaccine were likely to display complacency towards MPOX vaccines. Undergraduate Egyptian HCWs and those with prior knowledge about the MPOX vaccine were more likely to perceive constraints in MPOX vaccination. Female participants and those with a diploma or postgraduate education were more likely to engage in the calculation domain. In addition, HCWs with a diploma or postgraduate education were more likely to express a sense of collective responsibility toward vaccination.

However, previous findings have shown that younger females and those with higher education were more willing to receive the MPOX vaccine [36, 37]. These results suggest that specific groups of HCWs may be more hesitant towards MPOX vaccination, such as those from lower income levels, those with lower education levels, and male doctors.

Vaccination attitudes among HCWs can be influenced by various underlying factors. Cultural perceptions, healthcare policies, and education around vaccines in different regions play a significant role in shaping these attitudes [39]. Contextual factors, such as current events and media portrayal of vaccines, also impact perceptions and attitudes toward vaccination [39, 40]. Studies have shown that vaccine attitudes are influenced by demographic and ideological factors. For example, perceptions of vaccine risk can differ among individuals of different ethnic backgrounds, and there is a positive correlation between socioeconomic status and VH [41]. Immigrant parents may have negative attitudes or perceptions toward vaccination due to cultural values and misconceptions [42].

HCWs’ intentions to vaccinate are related to their knowledge, beliefs, and attitudes. Higher awareness, beliefs aligned with scientific evidence, and favorable attitudes towards vaccination are associated with greater intentions to vaccinate among healthcare workers [43]. Factors such as age, sex, profession, concerns about vaccine safety, fear of COVID-19, trust in government measures, and previous vaccination history can also influence HCWs’ attitudes toward COVID-19 vaccination [44].

Attitudes towards vaccination can also be influenced by personal factors. For example, nursing students’ attitudes towards the COVID-19 vaccine are influenced by family economic conditions, vaccination status of family members, and personal experiences with side effects from other vaccines [45]. HCWs have their views and concerns about the COVID-19 vaccine, and policymakers should consider these factors when planning vaccination campaigns [46].

Targeted campaigns and interventions could be developed to address these groups’ concerns and barriers to increase their confidence in MPOX vaccination [29]. Furthermore, highlighting the importance of vaccination to protect vulnerable individuals in the community, such as immunocompromised individuals, may help to increase collective responsibility and a sense of duty among HCWs to receive MPOX vaccination. This messaging could be included in educational and awareness-raising campaigns to boost vaccine acceptance among HCWs [29].

### Strengths and limitations

The use of the validated 5C scale questionnaire increases the reliability of the study. The 5C scale questionnaire aligns directly with the constructs and variables central to the research question, making it highly relevant to the topic under investigation. Also, the scale has demonstrated robust psychometric properties, including reliability and validity, in previous research. This ensures consistent measurement of the intended constructs and accurate capture of participants’ responses. To the best of our knowledge, this is the first study to examine the psychological aspects that affect Egyptian HCWs’ attitudes toward the MPOX vaccine.

The non-random sampling technique is one of the limitations of this study, which may limit the generalizability of the results. The study employed convenience and snowballing sampling methods due to specific constraints and limitations that made random sampling unfeasible. The limitations of the non-randomized sampling method should be acknowledged, as it may not fully represent the broader population of interest. The characteristics and perspectives of the participants may differ from non-participants, leading to potential biases in the results. Furthermore, the cross-sectional design made it impossible to determine causation and may have caused bias in reporting the findings. Other restrictions include the study’s subjective assessment of participants’ financial situations and the over-representation of women. Another limitation is the lack of information on the type of health professions and years of expertise, which are crucial factors in selecting whether to get immunized. Professional training, exposure to scientific evidence, and practical experiences can shape their attitudes and beliefs.

## Conclusions

This study highlights the importance of addressing various factors that can increase vaccine uptake and prevent the spread of MPOX among Egyptian HCWs. Efforts to contain MPOX should promote protective measures, such as vaccination, among high-risk groups like HCWs. Proper intervention measures should be implemented, including addressing different constraints and raising awareness of the importance of vaccination and its safety. These interventions may include tailored educational campaigns that address the specific concerns and misinformation prevalent among the MPOX community. Additionally, collaborations with trusted community leaders and influencers could be established to disseminate accurate information and promote vaccine confidence. Targeting HCWs can significantly curb the spread of infection and influence the wider community’s attitude towards vaccination. Vaccine recommendations from HCWs can potentially hinder community transmission of the virus. Future research can further advance our understanding of MPOX VH. This may include studies focusing on the impact of social media campaigns targeted at the MPOX community, exploring the effectiveness of peer-to-peer interventions, and examining the role of community-based organizations in promoting vaccine acceptance. By prioritizing these research areas, we can guide researchers in generating evidence that can inform the development and refinement of interventions tailored to the MPOX vaccination.

## Supplementary Information

Supplementary Material 1.

## Data Availability

For obtaining the data, please contact Dr. Ramy Ghazy ram_ghazy@alexu.edu.eg.
