# Supplementary material for: The intention of Egyptian healthcare workers to take the monkeypox vaccine: is urgent action required?
Source: BMC Health Serv Res. 2024 Oct 8;24:1204. doi: 10.1186/s12913-024-11147-0 (PMC11462741; doi:10.1186/s12913-024-11147-0)
Supplement: Supplementary file 1 — Supplementary Material 1. [file 12913_2024_11147_MOESM1_ESM.docx]

**The intension of Egyptian health care workers to take the monkey pox vaccine**

**Part1**: **Socio-demographic characters of the Egyptian HCWs:**

1. **Age (years): ………………………………**
2. **Gender:** Male Female
3. **Residence:** Rural Urban
4. **Marital status:** Single Married Widow Having partner
5. **Income level:** Low Middle High
6. **Educational level:** Precollege/High school Undergraduate (Bachelor) Diploma Post graduate (master) Post graduate (PhD)
7. **Having chronic diseases:** Yes No
8. **Having monkey pox:** Yes No I don’t know
9. **Knowing anyone died from monkey pox:** Yes No I don't know
10. **Having any idea about various types of monkey pox vaccines:**

Yes No I don’t know

**Part II: 5 C Scale for assessing the Psychological Antecedents of Egyptian HCWs toward Monkey pox vaccine:**

| **Subscale** | **Strongly disagree** | **agree** | **Neutral** | **agree** | **Strongly**  **disagree** |
| --- | --- | --- | --- | --- | --- |
| **Confidence**  Q1: I am completely confident that vaccines are safe  Q2: Vaccinations are effective  Q3: Regarding vaccines, I am confident that public authorities decide in the best interest of the community |  |  |  |  |  |
| **Complacency**  Q4: Vaccination is unnecessary because vaccine-preventable diseases are not common anymore  Q5: My immune system is so strong; it also protects me against diseases  Q6: Vaccine-preventable diseases are not so severe that I should be vaccinated |  |  |  |  |  |
| **Constrains**  Q7: Everyday stress prevents me from being vaccinated  Q8: For me, it is inconvenient to be vaccinated  Q9: Visiting the doctor makes me feel uncomfortable; this keeps me from being vaccinated |  |  |  |  |  |
| **Calculation**  Q10: When I think about being vaccinated, I weigh its benefits and risks to make the best decision possible  Q11: For each and every vaccination, I closely consider whether it is useful for me  Q12: It is important for me to fully understand the topic of vaccination before I get vaccinated |  |  |  |  |  |
| **Collective responsibility**  Q13: When everyone else is vaccinated, I don’t have to be vaccinated, too.  Q14: I get vaccinated because I can also protect people with a weaker immune system  Q15: Vaccination is a collective action to prevent the spread of diseases |  |  |  |  |  |
